# Everybody's Page

**Published:** 1891-10-24

**Authors:** 


### EVERYBODY'S PAGE.

Tempted by the earnest solicitations of an enthusiastic friend, we committed ourselves recently to the care of the sea in a sailing boat. The water being rough gave an opportunity to our friend, who is of a mathematical turn of mind, to discuss the shape of the waves. Much learned disquisition led him to the conclusion that the shape is parabolic; equal physical torture and mental anguish led us, who do not pretend to mathematical accuracy, to the certainty of the character being diabolic.

It is alleged that the Minister of War in Italy has been compelled recently, owing to the frequency of suicides in the Italian army, to call a council for the discussion of the causes of this tendency to self-destruction and for devising remedies. It is not stated whether the suicidal mania is one of long standing, but it is hinted that soldiers who have been questioned as to the cause ascribe it to overwork and improper food. If this is the true cause the authorities can speedily find a remedy for an unfortunate state of affairs, the existence of which should never have been possible.

There are, no doubt, many advocates of an operation as the only cure of squint, yet treatment based upon the facts that squint is due to error of refraction, that binocular vision can be restored, and that squint can be cured by the use of carefully made and fitted spectacles without an operation has produced the best results. Dr. A. R. Baker, of Cleveland, maintains that if the squint is alternating, and the vision fairly equal in both eyes, it is seldom necessary to operate, and that if the squint is fixed in one eye, and the sight very defective, and no improvement occurs after a patient trial with lenses and covering the good eye, only a cosmetic result can be obtained.

When making a great effort to accomplish a desired end, it is advisable to have some knowledge of geography and circumstances. Thus a lady of considerable enthusiasm, when crossing the Atlantic recently, wanted to see the sun rise, as she described it, "like a bridegroom leaving his chamber and rejoicing like a giant to run his course." She rose at four a.m., selected a comfortable place on the promenade deck, and waited events. After sitting there for three hours, and no sun making its appearance, she thought it was time to give it up. Before going to her state-room she took a walk on deck, and found to her surprise that the sun had already risen on the other side of the ship. It is much to her credit that she took her disappointment most good-humouredly, and no one enjoyed more than herself relating the incident in question.

New antiseptics are constantly being introduced, and the renown of some will probably have but a short career. One of the latest is called Styracol. It is alleged to be of value internally in catarrhal conditions of the alimentary tracts.

Sea salt, damped with a little water, is said to be very efficacious in relieving the pain caused by stings from all insects, including bees and wasps. After it is rubbed in, especially if this is done at once, the pain and swelling quickly subside. The only drawback to this remedy is that you must be near the sea or a box of Tidman's sea-salt at the time you are stung; it has been our frequent misfortune on these occasions to be in close proximity to a gooseberry bush.

It is not a pleasant sensation to have any foreign substance in the nose, and the height of discomfort must be reached when a five-inch hair-pin asserts its right to take up its lodging there for several days. This seems to have been the happy experience of a young American lady who swallowed a hair-pin, and who, it is alleged, was told by three physicians that she was suffering from imagination. Undeterred by this and with as firm a conviction as ever that she was suffering from hair-pin, she called the following day on another physician who succeeded in extracting the offending pin.

According to the *New York Tribune*, an Italian has been studying the contamination of the surface of the streets of large cities from a hygienic point of view. The results of his researches will not be reassuring to ladies who wear dresses which trail on the ground. The only comfort they can glean is, that Naples, which has not an enviable notoriety for cleanliness, afforded him his chief opportunities for research. He there found that in some streets the mud and refuse was so rich in bacteria that the streets might be compared to sewage!

They have a Solomon in the Far West in the person of the Dean of New Westminster. One of his parishioners died leaving two sons the whole of his property. The will omitted to specify the portion of the estate which each son was to receive. So little did the sons trust one another that each feared the other would get it. Under these circumstances, they referred the matter to the Dean of New Westminster, and, for the purposes of the narrative, we will name the sons A and B. The Dean, after a short reflection, said, "I decide that A shall divide the property to the best of his ability into two equal parts, and that B shall then choose which of the two portions he would like to take, A receiving the other."

